# 突变型P53蛋白在肺腺癌中的表达及其临床意义

**DOI:** 10.3779/j.issn.1009-3419.2015.01.04

**Published:** 2015-01-20

**Authors:** 春安 卞, 忠佑 李, 有涛 许, 洁 王, 林 许, 洪兵 沈

**Affiliations:** 1 210009 南京，南京医科大学附属江苏省肿瘤医院胸外科，江苏省恶性肿瘤分子生物学及转化医学重点实验室 Department of Thoracic Surgery, Jiangsu Laboratory of Molecular and Translational Cancer Research, Nanjing Medical University Affiliated Cancer Hospital of Jiangsu Province, Nanjing 210009, China; 2 210048 南京，南京江北人民医院 Nanjing Jiangbei People's Hospital, Nanjing 210048, China; 3 210029 南京，南京医科大学公共卫生学院 Public Health College of Nanjing Medical University, Nanjing 210029, China

**Keywords:** *TP53*基因, 突变, 肺肿瘤, 预后, *TP53* gene, Mutation, Lung neoplasms, Prognosis

## Abstract

**背景与目的:**

突变型*TP53*基因不仅丧失了抑癌功能，其编码的突变型P53蛋白还能获得促进细胞增殖、抑制细胞凋亡等功能。目前*TP53*基因在肺腺癌中突变的临床意义尚不十分明确。本研究旨在探讨突变型P53蛋白在肺腺癌组织中的表达及其临床意义。

**方法:**

回顾性分析120例肺腺癌手术患者的临床病理资料，应用免疫组化法检测患者组织标本中突变型P53蛋白的表达，采用*χ*^2^检验分析突变型P53蛋白表达与各临床病理参数的关系，采用单因素生存分析及多因素生存分析法分析突变型P53蛋白表达与总生存期的关系。

**结果:**

突变型P53蛋白在肺腺癌组织中的表达率为63.7%，突变型P53蛋白的表达与肿瘤大小（*P*=0.041）及病理分期（*P*=0.025）有关。单因素生存分析提示肿瘤大小（*P*=0.031）、淋巴结转移（*P* < 0.001）、病理分期（*P* < 0.001）以及突变型P53蛋白的表达（*P*=0.038）与患者总生存期密切相关。多因素生存分析提示仅有淋巴结转移（*P*=0.014）是患者总生存期的独立影响因素。

**结论:**

*TP53*基因突变的肺腺癌患者预后较差，突变型P53蛋白可以作为预测患者预后的分子标志物。

## Abstract (en)

**Background and objective:**

P53 is a tumor protein that acts as a tumor suppressor. The mutation of P53 may cause loss of tumor suppressor functions and gain of functions favoring cellular proliferation and apoptosis inhibition. The clinical implications of the tumor protein *P53* gene (*TP53*) mutation in lung adenocarcinoma are indefinite. The aim of this study is to explore the clinical significance of the mutant P53 protein expression in lung adenocarcinoma tissues.

**Methods:**

The clinicopathological data of 120 lung adenocarcinoma patients who underwent surgery were retrospectively analyzed. The mutant P53 protein expression was detected using the immunohistochemical method. Furthermore, the relationship between the mutant P53 expression and the clinicopathological parameters was analyzed using the *Chi*-square test, whereas that between the mutant P53 expression and overall survival was estimated using the *Kaplan-Meier* method and *Cox* regression model.

**Results:**

The mutant P53 protein expression rate was 63.3%. Accordingly, the mutant P53 expression was significantly associated with tumor size (*P*=0.041) and clinicopathological stage (*P*=0.025). On the one hand, a univariate survival analysis showed that tumor size (*P*=0.031), lymph node metastasis (*P* < 0.001), clinicopathological stage (*P* < 0.001), and mutant P53 expression (*P*=0.038) were associated with overall survival. On the other hand, a multivariate survival analysis showed that lymph node metastasis (*P*=0.014) was an independent poor prognostic factor for overall survival.

**Conclusion:**

Patients with lung adenocarcinoma with mutant TP53 had a poor outcome. Accordingly, the mutant P53 protein may serve as a molecular prognosis marker for lung adenocarcinoma patients.

*TP53*基因作为一种非常经典和重要的抑癌基因，一直以来受到广泛的关注和研究^[1]^。其编码的P53蛋白是一种应激反应蛋白，可以通过调节基因转录来应对基因毒性应激、癌基因信号激活以及DNA损伤等不利因素。近来的全基因组关联研究（genome wide association study, GWAS）提示*TP53*基因是人类恶性肿瘤中突变频率最高的基因之一^[2]^。相对于其他抑癌基因突变后仅仅是丧失了抑癌功能，*TP53*基因突变有其独特的特性，即突变的基因获得了新的促癌功能（gain of function, GOF）^[3]^。目前已有许多有关*TP53*突变在非小细胞肺癌（non-small cell lung cancer, NSCLC）中作用的研究，但结论尚不一，而且单独对*TP53*突变在肺腺癌中作用的研究则更为少见^[4-7]^。虽然同为NSCLC，肺鳞癌和肺腺癌的基因突变谱是完全不同的，研究^[8-10]^发现*TP53*、*EGFR*、*KARS*、*ALK*、*PIK3CA*、*SMAD4*等基因是肺腺癌的常见的驱动基因，因此将肺鳞癌和肺腺癌分别研究更为合理。由于突变的*TP53*基因编码的P53蛋白半衰期较长，而野生型*TP53*基因编码的P53蛋白半衰期极短，所以突变型P53蛋白在细胞核内积累从而可以被免疫组化法检测，而野生型P53蛋白无法被免疫组化法检测到^[11]^。本研究利用免疫组化SP法检测突变型P53蛋白在肺腺癌组织中的表达情况，分析其与患者临床病理特征及预后的关系，探讨突变型P53蛋白在肺腺癌中的作用及其对预后的影响。

## 1 资料与方法

### 1.1 标本及病例资料来源

收集南京医科大学附属江苏省肿瘤医院2007年1月-2009年3月期间手术切除并经术后病理确诊为肺腺癌的石蜡标本120例。所有纳入研究的患者均有完整的病历及随访资料。随访期间死于其他疾病的患者不被纳入研究。所有患者接受的均为根治性手术，所有患者术前均未接受放疗或化疗。本研究经江苏省肿瘤医院医学伦理委员会批准。

### 1.2 免疫组化SP法测定突变型P53蛋白表达

鼠抗人突变型P53单克隆抗体购自Santa Cruz公司（克隆号clone SC126），SP试剂盒购自北京中山公司。染色方法采用常规SP法，突变型P53一抗工作浓度为抗体原液稀释1, 000倍。采用生物素标记的二抗进行染色。用已知的阳性胰腺癌组织作为阳性对照，使用PBS缓冲液代替一抗做为阴性对照。

### 1.3 免疫组化结果判定

突变型P53蛋白的表达主要定位于细胞核，以胞核中有棕褐色颗粒染色为阳性（图 1），每个切片于高倍镜下随意取5个不重复视野计数，计数所有细胞，表达强度按阳性细胞所占百分比进行判断，取其平均值，以阳性细胞数≤10%为阴性，阳性细胞数 > 10%为阳性。免疫组化的结果判定由两个高年资病理科医生共同作出。

**1:**
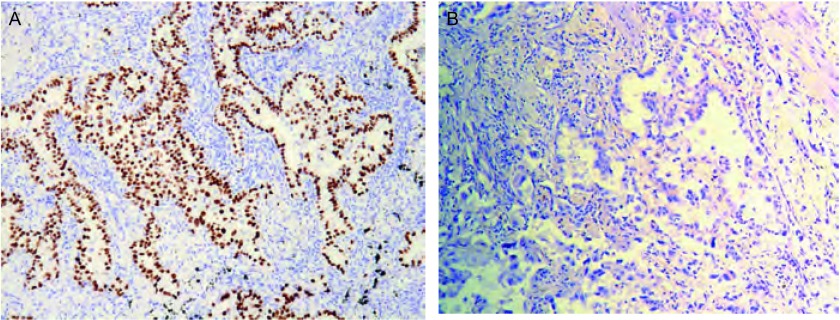
突变型P53在肺腺癌组织中的免疫组化（SP法，×100）。A：突变型P53阳性表达（细胞核棕黄色染色）；B：突变型P53阴性表达。 Mutant P53 expression in lung adenocarcinoma by immunochemistry (SP, ×100). A: Positive mutant P53 expression (staining was detected in nucleus); B: Negative mutant P53 expression.

### 1.4 统计学方法

实验数据应用SPSS 17.0统计软件进行统计分析，组间率的比较采用*χ*^2^检验，单因素生存分析采用*Kaplan-Meier*法（*Log-rank*检验），多因素生存分析采用*Cox*风险比例模型。以*P* < 0.05为差异有统计学意义。

## 2 结果

### 2.1 临床病理特征及预后

所有纳入研究的患者中男性58例，女性62例。手术时的平均年龄为59.4岁。截止到随访时间（2014年3月）有20.8%（25例）的患者仍存活。采用寿命表法统计出患者的1年、3年、5年的生存率分别为61%、39%、33%。肿瘤大小≤3 cm（T1）的占20%（24例）， > 3 cm≤7 cm（T2）的占60.8%（73例）， > 7 cm（T3、T4）的占19.2%（23例）。淋巴结转移的占40.8%（49例）。肿瘤高分化的占26.7%（32例）、中分化的占34.1%（41例）、低分化的占39.2%（47例）。仅有10.8%（13例）患者有胸膜侵犯。临床分期为Ⅰ期、Ⅱ期和Ⅲ期的患者分别占30.8%（37例）、39.2%（47例）和30.0%（36例）（表 1）。

**1:** 突变型P53的表达患者临床病理参数的关系 Mutant P53 expression in relation to clinicopathological parameters (*n*=120)

Characteristic	*n*	Mutant P53 expression		*χ*^2^	*P* value
		Negative	Positive		
Age (yr)					
≤60	73	26 (35.6%)	47 (64.4%)	0.089	0.766
> 60	47	18 (38.3%)	29 (61.7%)		
Gender					
Male	58	25 (43.1%)	33 (56.9%)	2.003	0.157
Female	62	19 (30.6%)	43 (69.4%)		
Tumor size (cm)					
T1 (≤3)	24	14 (58.3%)	10 (41.7%)	6.412	0.041
T2 (> 3≤7)	73	24 (32.9%)	49 (67.1%)		
T3 and T4 (> 7)	23	6 (26.1%)	17 (73.9%)		
Lymph nodes					
Negative	71	27 (38.0%)	44 (62.0%)	0.139	0.709
Positive	49	17 (34.7%)	32 (65.3%)		
Differentiation					
Well	32	16 (50.0%)	16 (50.0%)	3.341	0.188
Moderate	41	13 (31.7%）	28 (68.3%)		
Poor	47	15 (31.9%)	32 (68.1%)		
Pleural invasion					
Negative	107	39 (36.4%)	68 (63.6%)	0.020	0.887
Positive	13	5 (38.5%)	8 (61.5%)		
Stage					
Ⅰ	37	20 (54.1%）	17 (45.9%）	7.384	0.025
Ⅱ	47	15 (31.9%）	32 (68.1%）		
Ⅲ	36	9 (25.0%）	27 (75.0%）		

### 2.2 突变型P53蛋白的表达及其与临床病理特征的关系

免疫组化法检测突变型P53蛋白在肺腺癌组织中的表达率为63.3%（76/120）。卡方检验显示突变型P53蛋白的表达与患者肿瘤大小（*P*=0.041）和临床病理分期（*P*=0.025）有相关（图 1）。

### 2.3 突变型P53蛋白的表达及临床病理特征与预后的关系

运用*Kaplan-Meier*法对120例患者进行单因素生存分析发现，肿瘤的大小（*P*=0.031）、淋巴结转移（*P* < 0.001）、临床病理分期（*P* < 0.001）、突变型P53蛋白的表达（*P*=0.038）与患者的总生存期密切相关（图 2）。对单因素分析中*P* < 0.05的因素进一步进行*Cox*风险比例模型多因素，结果显示仅有淋巴结转移（*P*=0.014）为肺腺癌总体生存期的独立预后因素（表 2）。

**2:**
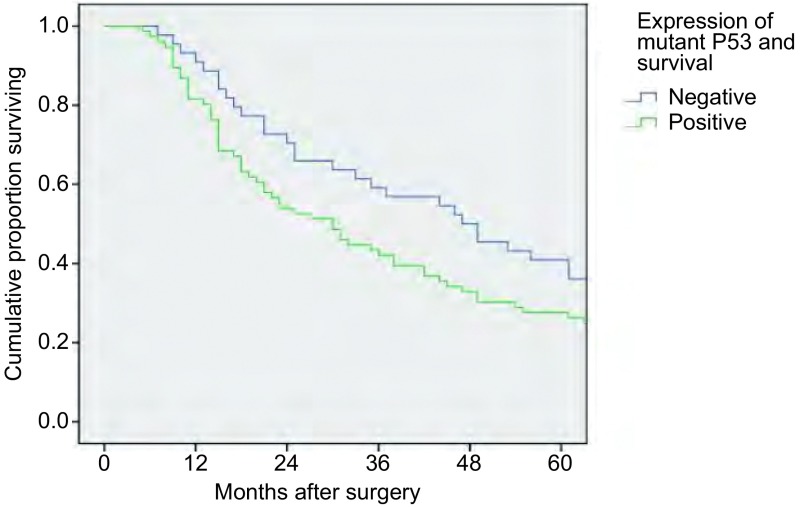
突变型P53蛋白表达与总生存期的生存曲线 The overall survival curve of patients according to mutant P53 expression

**2:** 患者总生存期的单因素及多因素分析 Univariate and multivariate analyses of overall survival (*n*=120)

Characteristic	Univariate analysis			Mutivariate analysis	
	Median (mo)	*P* value (*Log-rank*)		Hazard ratio (95%CI)	*P* value
Age (yr)				-	-
≤60	37.000	0.883			
> 60	30.000				
Gender				-	-
Male	36.000	0.917			
Female	30.000				
Tumor size (cm)					
T1 (≤3)	45	0.031		1.423 (0.990-2.044)	0.056
T2 (＞3≤7)	35				
T3 and T4 (> 7)	15				
Lymph nodes					
Negative	49	< 0.001		2.222 (1.172-4.210)	0.014
Positive	17				
Differentiation				-	-
Well	49	0.067			
Moderate	35				
Poor	21				
Pleural invasion				-	-
Negative	37	0.091			
Positive	23				
Stage					
Ⅰ	55	< 0.001		1.075 (0.706-1.636)	0.735
Ⅱ	30				
Ⅲ	16				
P53 expression					
Negative	47	0.038		1.320 (0.834-2.091)	0.236
Positive	30				

## 3 讨论

肺癌是当今世界上发病率和死亡率最高的恶性肿瘤之一^[12]^，并且流行病学统计发现肺癌中的肺腺癌发病率越来越高，目前已经取代肺鳞癌成为发病率最高的NSCLC^[13]^。长期以来，以肺鳞癌和肺腺癌为代表的NSCLC的预后无明显改善，直到近十年来，随着分子生物学的进展，EGFR、KARS、TP53等一批肺腺癌驱动基因被陆续发现^[14]^，导致一些靶向治疗药物的出现，极大地改善了肺腺癌患者的预后^[15, 16]^。人类*TP53*基因定位于17号染色体p13，全长16 kb-20 kb，含有11个外显子，转录2.8 kb的mRNA，编码蛋白质为P53，是一种核内磷酸化蛋白。*TP53*是迄今为止发现的与人类肿瘤相关性最高的基因。过去一直把它当成一种癌基因，直至1989年才发现起癌基因作用的是突变的P53，后来证实野生型*P53*是一种抑癌基因，在正常情况下，细胞中野生型P53蛋白的含量很低，且半衰期极短，所以很难检测出来。一直以来，*TP53*基因都是生物学界研究的热点，有关野生型的*TP53*基因的抑癌作用和突变型的TP53的致癌功能，已经得到公认。众多临床证据^[17]^也显示*TP53*基因突变与多种恶性肿瘤的不良预后有关。尽管如此，突变的TP53在肺癌中的意义尚不明确。Ding等^[18]^发现45%的肺腺癌患者的*TP53*基因存在突变，而本研究中肺腺癌组织中测得的*TP53*突变率为63.3%，较其报道的突变率略高。Ahn等^[11]^报道*TP53*的突变与NSCLC的预后无关。而另一些报道^[19-22]^则认为*TP53*突变是NSCLC的不良预后标志。本研究中亦发现*TP53*的突变与患者不良的总生存期预后有关，但并不是独立预后因素，猜测可能是因*TP53*基因是一个关键基因，调控多种基因的转录表达、作用及机制较为多样复杂所致。另外，有报道^[23, 24]^在动物实验中发现含有*TP53*突变的小鼠肿瘤更具侵袭性更容易转移。但本研究中未发现*TP53*突变与肿瘤转移相关，仅与肿瘤的大小及临床病理分期相关。对于肺癌中*TP53*突变的作用各个研究得出的结论不一，我们认为可能与分子的异质性、*TP53*突变的各种不同基因型以及实验采用的不同方法、不同分组、样本量的大小不同有关。故今后需要进行更大样本量的实验，更加完善的实验方法，以取得较为可靠一致的结论。

综上所述，本研究应用免疫组化法对120例肺腺癌患者的突变型P53蛋白进行了检测，发现其与患者的肿瘤大小，临床病理分期及总生存期有关。本研究结果提示TP53突变是肺腺癌患者不良预后的一个重要因素，未来值得对相关机制进一步进行研究。
